# The proprioceptive puzzle: An observational study investigating the effects of cervical proprioceptive errors on quantitative sensory testing and body awareness in young individuals

**DOI:** 10.1371/journal.pone.0321645

**Published:** 2025-04-21

**Authors:** Nagihan Acet, Sena Begen

**Affiliations:** Department of Physiotherapy and Rehabilitation, Faculty of Health Sciences, Atılım University, Ankara, Türkiye; Umm Al-Qura University, SAUDI ARABIA

## Abstract

**Objective:**

The present study investigates the effects of cervical proprioceptive errors (CPE) on body awareness and quantitative sensory testing (QST), including the pressure pain threshold, temporal summation, and conditioned pain modulation in young individuals.

**Materials and methods:**

Included in this prospective cross-sectional study were 78 participants who were divided into two groups based on the presence or absence of CPE. The study was registered on ClinicalTrials.gov with the clinical trial number [NCT06559397]. Cervical proprioception was measured using the “head position error test”, body awareness was assessed using the “Body Awareness Questionnaire”, QST was assessed using a mechanical pressure algometer, and conditioned pain modulation was evaluated using cold stimulus.

**Results:**

The study revealed a significant reduction in body awareness among those with CPE (p < 0.001), while no significant differences were found between the groups in terms of QST, including the pressure pain threshold, temporal summation, and conditioned pain modulation (p > 0.05).

**Conclusions:**

CPE can have a significant impact on body awareness, leading to a decrease in the ability to perceive one’s own body. While the present study offers no significant findings related to QST, it provides new insights into the relationship between proprioception, body awareness, and pain processing mechanisms. Clinically, the results suggest the importance of integrating interventions aimed at enhancing body awareness into the treatment protocols of patients with CPE.

## Introduction

“Proprioception” refers to the ability of a person to determine the position of their body and its segments based on information received from receptors in the joints, muscles and tendons. Previous studies have suggested that pain alters proprioception, and although it is frequently linked to injury or pain, it remains unclear whether proprioceptive deficits are a cause or outcome of pain.

Previous studies have focused primarily on proprioceptive errors in the extremities, while spinal proprioception has been overlooked, despite its critical role in postural stability [1]. The cervical region is a primary source of proprioceptive information due to the high density of mechanoreceptors in the region [2]. The suboccipital region contains 200 muscle spindles per gram of muscle tissue, compared to just 16 per gram in the first lumbrical muscle of the thumb [3]. Cervical muscle spindles continuously relay information to the central nervous system (CNS), which then regulates muscle activation accordingly [4]. Moreover, these receptors are also connected to the vestibular, and visual system [5,6], and provide information essential for the reflexes controlling head, eye and postural stability [7].

Cervical proprioceptive errors (CPE), even in the absence of injury or pain, can influence central pain-related parameters via the connection between proprioception and the CNS. The Quantitative Sensory Test (QST) is a psychophysical tool for assessing pain perception that is widely used for the evaluation of both peripheral and central sensitisation [8–10]. Among the most commonly applied QST measurements for the diagnosis of changes in pain processing mechanisms are those measuring the pressure pain threshold, temporal summation and conditioned pain modulation [11].

The ***pain threshold*** is the point at which a sensation becomes painful [11], while ***temporal summation*** refers to the gradual increase in pain with repeated stimuli [12]. Measurements of ***conditioned pain modulation****, as* one of the primary mechanisms behind endogenous analgesia, assess the potential to reduce pain perception through the application of mechanical, electrical, thermal or cold stimulus to different areas. Such tests can provide insights into the functioning of A and C fibres, as well as their central pathways [11,13], as well as valuable insights into individual pain mechanisms. While the role of proprioception in central processing is crucial, its effects on central pain mechanisms remain unclear, and there have been no studies to date exploring the impact of CPE on QST.

“Body awareness” has attracted increased interest recently in health-related scientific studies, referring to an individual’s awareness of the different parts and dimensions of their bodies. From a neuroscientific perspective, it refers to the brain’s interpretation of signals from the body and the environment related to one’s body, movements and surroundings. These signals gradually integrate over time, creating bodily experiences that significantly influence one’s self-perception, surroundings and social interactions [14]. While the central processing of proprioceptive information has been linked to body awareness, the impact of CPE on body awareness remains unexamined.

The present study aimed to investigate the impact of CPE on body awareness and QST parameters in young individuals.

## Materials and methods

This prospective, cross-sectional study was written using the STROBE checklist and was approved by the Atılım University Ethics Committee (Number: 604.01.02–159/ date: 24.01.2024). Written informed consent was obtained from all participants in the study. The study was conducted per the principles stated in the Declaration of Helsinki. After providing information about the study to the participants, those who agreed to participate were included upon obtaining their written consent.

An invitation to participate in the study was posted on the announcement page of Atılım University in the form of a recruitment flyer containing information about the study, and participants were recruited from among the staff located on the Atılım University campus between 01/09/2024 and 30/10/2024, using a non-random convenience sampling method. The respondents were then evaluated face-to-face by a Research Assistant in the research laboratory of the Department of Physiotherapy and Rehabilitation on the campus, and those who met the inclusion criteria and who agreed to take part were included in the study.

**Fig1:**
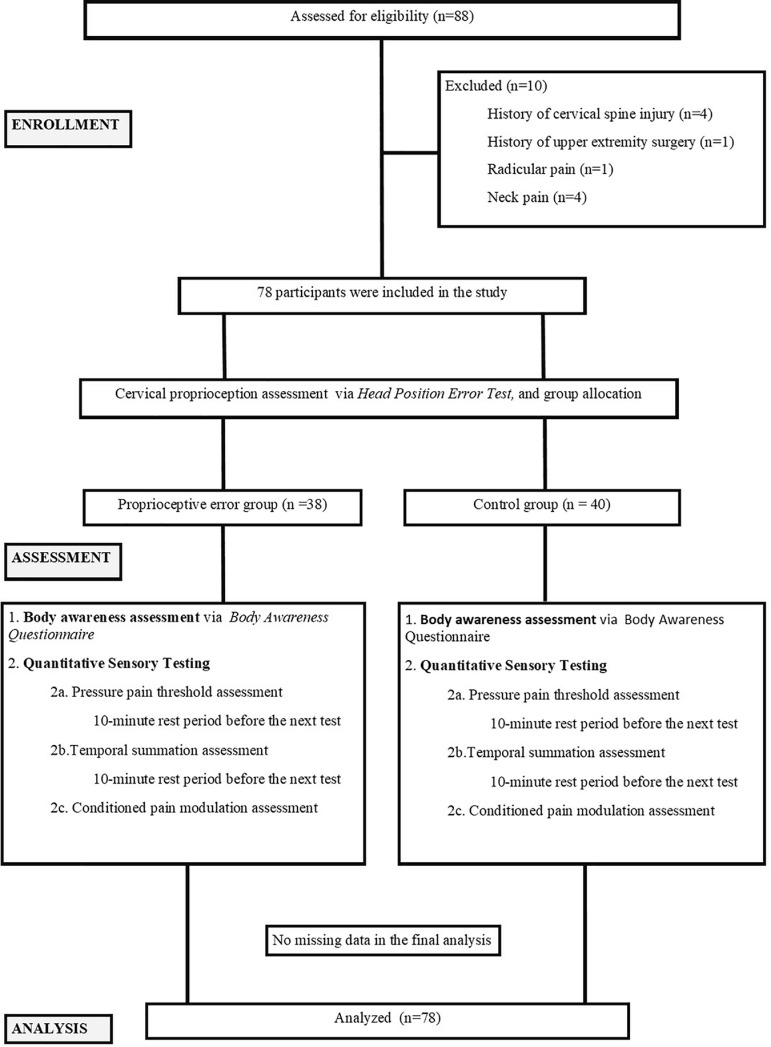
Flow chart.

A flowchart delineating the participant selection and group allocation processes applied in the study is presented in Fig 1. [Flow chart]. A total of 88 respondents were initially assessed for eligibility, of whom 10 were excluded based on the predefined criteria: history of cervical spine injury (n=4), history of upper extremity surgery (n=1), presence of radicular pain (n=1) and current neck pain (n=4). Consequently, 78 participants met the inclusion criteria and were enrolled in the study. There were no deviations from the study protocol as originally planned. No adverse events or unintended effects were observed during the study.

Respondents aged 18–25 who voluntarily agreed to participate were included in the study. Each respondent completed the SF-12 Health Survey – a simple and reliable 12-item questionnaire used to assess overall well-being that generates two main scores: the physical component and the mental component summary. Final scores are in the range of 12–111, with higher scores indicating better health and well-being, and those with an SF-12 Health Survey score of 50 or above were included in the present study. The SF-12 Health Survey can be considered a valuable tool for the evaluation of general health status in both clinical and research settings [15,16].

Exclusion criteria were sensory loss, neck pain, radicular pain, a history of cervical or upper extremity surgery, a history of cervical trauma, the presence of neurological disease, musculoskeletal injury, vestibular pathology, or cold allergy.

The participants (unit of assignment: individual) were divided into two groups based on the presence of proprioceptive error (cut-off value: >5°): the ***proprioceptive error group*** (n=38) (those with a CPE greater than 5 degrees in at least one direction), and the ***control group*** (those with a CPE less than 5 degrees) (n=40), rather than being randomly allocated. No blocking, stratification, or minimization techniques were applied. Both groups underwent comprehensive assessments that included body awareness evaluations and quantitative sensory testing, which were determined as the primary outcomes. The assessments were administered face-to-face once after obtaining informed consent. The evaluation duration for each patient was approximately 30–40 minutes.

In both groups, body awareness was assessed as the primary outcome using the “Body Awareness Questionnaire”, while QST, including pressure pain threshold and temporal summation, were measured bilaterally using an algometer (2 cm lateral to the C2 spinous processes and the midpoint of the upper part of the trapezius muscle).

### Data collection

The participants’ height, weight, and body mass index (BMI) were measured by the researcher using standardized assessment tools to ensure accuracy and consistency, while age was recorded based on self-reported information. QST and body awareness assessment were conducted by the other researcher involved in the study, who was unaware of whether the participant had a proprioceptive deficit. This ensured evaluator blinding and minimized potential bias.

#### Assessment of cervical proprioception.

The “head position error test” was used to assess cervical proprioception and based on the presence of CPE (cut-off value: >5°), the participants were divided into two groups: the Proprioceptive error (experimental) group (those with CPEs greater than 5 degrees) and the Control group (those with CPEs of less than 5 degrees). Participants with errors greater than 5 degrees in at least one direction were considered to have a proprioceptive deficit and were included in the experimental group.

The head position error test was performed while the patient was seated including a right and left rotation using a CROM device [17]. The maximum cervical range of motion was determined, and the midpoint of the maximum range was identified as the “target position”. The participants were instructed to close their eyes, after which their heads were passively moved to the target position by the physiotherapist, held there for 3 seconds, and then returned to the neutral position. The participants were then asked to actively replicate the same target position, and to repeat the movement six times. The differences between the target position and the achieved positions were recorded, and the averages were calculated, and errors greater than 5 degrees were recorded as a CPE.

#### Assessment of body awareness.

Body awareness was assessed using the Body Awareness Questionnaire (BAQ). The Turkish version of the questionnaire was used in our study [18]. It is an 18-item questionnaire divided into four subgroups, measuring 1. Changes in bodily processes, 2. Sleep-wake cycle, 3. Prediction of illness onset, and 4. Prediction of bodily responses. The questionnaire aimed to determine whether the participant’s body composition sensitivity was normal or abnormal. consists of 18 items, with a minimum score of 18 and a maximum score of 126. The participants were asked to rate each item on a scale of 1–7 and the scores were totalled, with higher scores indicating better body awareness. The validity and reliability of the questionnaire were reported to be high [18,19].

#### Quantitative sensory testing (QST).

***Assessment of pressure pain threshold:*** A pressure algometer (Baseline Force Gauge Model 12–0304; Baseline, NY, USA) was used to assess the pressure pain threshold. The patient was seated, and pressure was applied perpendicularly and bilaterally using the algometer to different points in the cervical region (2 cm lateral to the C2 spinous processes, at the midpoint of the upper part of the trapezius muscle) at a rate of approximately 3 N/s over an area of 0.5 cm². The participant was asked to report the moment they first felt discomfort as the pressure was applied, and the pressure value at this moment was recorded from the device as the “pressure pain threshold” and noted on the test protocol. A second measurement was taken at each region after an interval, the average of the two measurements was noted.

***Assessment of temporal summation:*** Ten minutes after measuring the pressure pain threshold, a temporal summation assessment was conducted bilaterally at the same points (2 cm lateral to the C2, at the midpoint of the upper part of the trapezius muscle) in which pressure was applied matching the participant’s pressure pain threshold (the point at which discomfort was first felt) 10 times in succession. The participant was again asked to identify the point at which they first felt discomfort (final threshold), and the difference between the initial threshold and the final threshold was recorded as the Temporal Summation Value [20]

***Assessment of conditioned pain modulation:*** The first step in the assessment of conditioned pain modulation involved the application of increasing pressure to the right trapezius muscle using an algometer until the participant reported a pain level of 4/10 on the Visual Analog Scale. The participant was asked to say “stop” when the pain level 4 was reached, at which point the stimulus was terminated. Five minutes after the test stimulus, the participant was asked to immerse their right hand in ice water for 20 seconds as the conditioned stimulus. For each immersion, 3 litres of fresh tap water was poured into a glass container and adjusted to 7°C using ice cubes, verified with a thermometer. Immediately after the participant removed their hand from the water, the same pressure that produced a 4/10 pain level on the right trapezius was applied to the left trapezius using the algometer, and the participant was asked to rate the pain again. For the participants who were unable to keep their hand in the water for 20 seconds, the assessment was conducted immediately after they removed their hand. The ratio of pain scores before and after the conditioned stimulus was multiplied by 100 and recorded as the final value [21,22].

#### Sample size calculation.

The sample size calculation was conducted using G*Power to determine the statistical power of the study. A post hoc power analysis was performed under the t-test family for a Wilcoxon-Mann-Whitney test (two groups) to evaluate group differences in body awareness questionnaire scores (outcome variable). The effect size was calculated as 1.107, based on the pooled standard deviation (15.41) and the mean values of Group 1 (74.16) and Group 2 (91.22). A two-tailed test was chosen to allow for bidirectional hypotheses. The alpha error probability (α) was set to 0.05, and the sample sizes were 40 participants in Group 1 and 38 participants in Group 2, reflecting a slightly unequal sample size ratio (n1 ≠ n2). The achieved statistical power (1-β) was calculated as 0.99, indicating a very high probability of detecting a true effect given the sample size and effect size parameters.

### Statistical analysis

The statistical analyses were conducted using IBM SPSS Statistics (Version 23.0. Armonk, NY: IBM Corp.) The unit of analysis was the group. As analyses were conducted at the group level, no further adjustments were needed. Descriptive statistics of categorical variables were given using numbers and percentages. The Chi-square test was used to compare categorical variables between groups. The normality assumptions of numerical variables were assessed using the Shapiro-Wilk test along with skewness and kurtosis values. A skewness and kurtosis range of ±1 was considered acceptable for normal distribution. For between-group comparisons, the independent samples t-test was used for normally distributed data, and results were reported as mean ± standard deviation, t value, 95% confidence interval, and p-value. For non-normally distributed data, the Mann-Whitney U test was performed, and results were presented as mean ± standard deviation, median (1st and 3rd quartiles), mean rank, Z value, and p-value. All statistical analyses were conducted at a significance level of 0.05, and two-tailed tests were applied to assess differences between groups. There were no missing data in this study. An intention-to-treat analysis approach was applied, and all participants were analyzed according to their initial group allocation. All analyses were conducted as pre-specified, and no additional subgroup or exploratory analyses were performed. No direct comparison with the broader target population was conducted.

## Results

Table 1 presents the distribution of proprioceptive error types between groups and the comparison of gender distribution. There was no significant difference in gender distribution between the groups (p = 0.687, χ² = 0.162).

**Table 1:** Proprioceptive error types between groups and comparison of gender distribute.

		Proprioceptive error group	Control group	Total	p-value ^χ²^ χ² (df = 1)
**n (%)**		38 (48.7%)	40 (51.3%)	78 (100%)	
**Proprioceptive Error Type**	**Only right rotation**	9 (11.5%)	0 (0%)	9 (11.5%)	
	**Only left rotation**	13 (16.6%)	0 (0%)	13 (16.6%)	
	**Bidirectional rotation**	16 (20.5%)	0 (0%)	16 (20.5%)	
	**No error**	0 (0%)	40 (51.3%)	40 (51.3%)	
**Gender**	**Female**	24 (63.2%)	27 (67.5%)	51 (65.4%)	0.687 0.162 ..
	**Male**	14 (36.8%)	13 (32.5%)	27 (34.6%)	

χ²: Chi-Square test

The demographic characteristics of the participants, including age, height, weight and body mass index (BMI), are summarised in “Table 2” revealing no statistically significant differences between the proprioceptive error group and the control group (p>0.05).

**Table 2:** Demographic characteristics of the participants.

	Proprioceptive Error Group (n = 38)	Control Group (n = 40)	F	t	Mean difference	95% Confidence Interval of Difference		Cohen’s d	p -value ^t^
	$\bar{\text{x}}$ ± sd (min-max)	$\bar{\text{x}}$ ± sd (min-max)							
						Lower	Upper		
**Age** **(year)**	22.37 ± 1.06 (20-25)	22.60 ± 1.59 (20-30)	0.57	0.71	0.23	-0.38	0.84	-0.16	0.45
**Height (cm)**	1.69 ± 0.09 (1.54-1.90)	1.71 ± 0.85 (1.53-1.92)	1.13	0.61	0.01	-0.02	0.05	-0.03	0.53
**Weight (kg)**	66.86 ± 13.16 (43-94)	65.72 ± 12.18 (46-105)	1.32	-0.39	-1.13	-6.81	4.53	0.09	0.69
**BMI (kg/m**^**2**^)	22.96 ± 3.06 (17.22-30.84)	22.36 ± 3.02 (17.3-32.41)	0.01	-0.87	-0.60	-1.96	0.76	0.19	0.38

^t^: Independent T test; BMI: Body mass index; cm: centimetre; kg: kilogramme; kg/m^2^: kilogramme/ metre^2^

Table 3 presents a comparative between-group analysis of proprioceptive errors, body awareness and QST. An analysis of the data reveals the body awareness of the proprioceptive error group to be lower than in the control group, to a statistically significant degree (p<0.001), indicating a link between CPE and decreased body awareness. In contrast, no statistically significant differences were noted between the groups in the QST findings, including the pressure pain threshold, temporal summation and Conditioned Pain Modulation values (all p > 0.05).

**Table 3:** Between-group comparison of proprioceptive errors, body awareness, and pain-related parameters.

	Proprioceptive Error Group (n=38)			Control group (n=40)			Mean Difference	F	t	Cohen’s d	p value^t^
	$\bar{\text{x}}$ ± sd (min-max)			$\bar{\text{x}}$ ± sd (min-max)							
**Proprioceptive error-left rotation** **(Degree)**	5.99±2.76 (0.33-12.83)			1.92±1.15 (0-4)			-4.07	9.86	-8.56	2.10	**<0.001** ^*^
	$\bar{\text{x}}$ **± sd** **(min-max)**	**Median** **(Q1-Q3)**	**Mean rank**	$\bar{\text{x}}$ **± sd** **(min-max)**	**Median** **(Q1-Q3)**	**Mean rank**	**Mean Difference**	**Median Difference**	**U/ Z**	**r**	**p value** ^u^
**Proprioceptive error- right rotation (Degree)**	4.64±1.54 (0-7.83)	5 (4.12-5.66)	56.45	1.67±1.23 (0-3.83)	1.33 (0.83-3)	23.40	-2.69	-3.67	116/-6.44	0.73	**<0.001** ^*^
**Body awareness**	74.15±15.22 (26-112)	71 (67-83)	28.12	91.22±15.38 (55-114)	92 (82.25- 103.50)	50.31	17.06	21	327.50/ -4.33	-0.49	**<0.001** ^*^
**C2 pressure threshold**	3.32±1.05 (1.85-5.7)	3.22 (2.46-4.03)	44.04	3.02±1.28 (1.55-8.5)	2.67 (2.25-3.40)	35.12	-0.30	-0.55	587.50/-1.72	-0.19	0.84
**Upper trapezius pressure threshold**	4.12±1.56 (1.50-7.80)	3.75 (3-4.62)	41.11	3.87±1.26 (1.75-6.75)	3.57 (2.75-4.72)	37.98	-0.25	-0.18	699/-0.61	-0.07	0.55
**C2 Temporal summation**	2.80±1.99 (0.1-9.35)	2.37 (1.68-4.02)	40.55	2.42±1.13 (0.20-5.25)	2.40 (1.70-3.03)	38.50	-0.38	0.02	720.00/ -0.40	-0.05	0.68
**Upper trapezius temporal summation**	3.62±2.61 (0.20-10.80)	3 (1.60-6.16)	37.32	3.96±2.20 (0.25-10.50)	3.55 (2.81-5)	41.58	0.33	0.54	677.00/-0.83	-0.09	0.40
**Conditioned pain modulation**	2±2.22 (-3-7)	1.50 (0.50-4)	42.59	1.19±1.58 (-4.40-5.10)	1 (0.50-2)	36.56	-0.80	-0.50	642.50/ -1.18	-0.13	0.23

^t^: Independent Samples t-Test;

^u^: Mann-Whitney U Test,

*:p=<0.05

## Discussion

The present study investigates the impact of CPE on body awareness and QST, including the pressure pain threshold, temporal summation and conditioned pain modulation in a sample of young asymptomatic individuals, and is, to the best of our knowledge, the first study to date to examine the effects of CPE on body awareness and QST. Our findings reveal a significantly lower level of body awareness in those with CPE, while no significant differences were noted between the groups in the QST findings. This suggests that while CPE may not have a pronounced impact on central pain processing mechanisms, such errors may play a crucial role in the self-perception and awareness of their own bodies.

A total of 78 participants (51 females, 65.4%; 27 males, 34.6%) were included in the study. In the proprioceptive error group, 27 participants (52.9%) were female, whereas female constituted 51.9% of the control group, indicating comparable gender distribution between the groups. The number of studies examining the relationship between cervical proprioceptive errors and gender in healthy individuals is limited in the literature, highlighting the need for further research in this area.

When the sample was analyzed based on the presence of proprioceptive error, 51.3% of the participants exhibited deviations exceeding 5 degrees in at least one direction. A 2024 study, investigating cervical joint position sense errors in 400 young individuals, reported right rotation errors in 64.3% and left rotation errors in 62.5% of the participants [23]. While the cutoff value was set at 4.5 degrees in the referenced study, our study used a threshold of 5 degrees. A review of studies on cervical proprioceptive errors in the literature reveals that research on healthy individuals is limited. Moreover, cervical proprioception has predominantly been studied in the presence of pain [24–33]. Given that most studies on cervical proprioception have focused on individuals with pain, there remains a need for further research to elucidate its characteristics in healthy populations.

The effect of CPE on body awareness was investigated in the present study, revealing that such errors significantly reduced body awareness. To the best of our knowledge, this is the first study to date to investigate the impact of CPE on body awareness, being a crucial factor that influences the ability of the individual to perceive their bodies and environments and to act accordingly. In clinical practice, high body awareness is essential for the maintenance of postural stability, improved motor control and optimal functional movements [34,35]. Such an ability helps reduce the risk of injury in daily life and in athletic activities. High body awareness minimises the stress and strain imposed on the musculoskeletal system by ensuring proper positioning and movement control. Enhancing the body awareness of those undergoing rehabilitation can accelerate recovery and reduce the risk of re-injury.

Although the effects of proprioceptive errors on body awareness have not been previously addressed in the literature, a limited number of studies have examined the relationship between body awareness and factors indirectly related to proprioception in different populations. A 2023 study investigated the relationship between body awareness, physical activity, and strength levels in young athletes. The study, conducted with 76 athletes, reported that as total physical activity scores increased, body awareness levels also improved. However, no clear relationship was established between strength and body awareness [36]. Another study investigated the effects of body awareness on trunk control, upper extremity function, fear of falling, balance, functional level, and independence in 35 stroke patients. The findings indicated that body awareness influenced all the examined parameters [37]. In a study conducted by Baskan, the impact of chronic musculoskeletal pain and cognitive impairment on body awareness was assessed in 265 individuals aged 65 and older. A moderate positive correlation was found between cognitive status and body awareness, while a weak negative correlation was observed between pain and body awareness [38]. Considering these three studies, the parameters examined—strength (Study 1), trunk control, function, and fear of falling (Study 2), and cognitive status and pain (Study 3)—are indirectly related to proprioception, providing crucial evidence for the body awareness-proprioception relationship. However, the present study is the first in the literature to explore this relationship explicitly, making a significant contribution to the proprioceptive puzzle.

QST plays a critical role in assessments of pain perception. It is widely used to determine the presence of peripheral and central sensitization. It can be considered a non-invasive and reliable approach to the assessment of both sensory function loss and gain, thus contributing to our understanding of underlying pathophysiological mechanisms [11]. In QST, the perceptual responses of the patient to systematically applied and quantifiable sensory stimuli are measured, providing insights into somatosensory function or dysfunction [11]. By evaluating the integrity of the entire neural axis-from receptor to brain-QST complements clinical neurophysiological studies that typically focus on large sensory fibres. While QST cannot identify the precise source of somatosensory dysfunction, it offers valuable information on the function of large myelinated A-beta fibres, thinly myelinated A-delta fibres and small unmyelinated C fibres, as well as their corresponding central pathways [11]. The tests applied in the present study include those measuring the pressure pain threshold, temporal summation, and conditioned pain modulation.

The pressure pain threshold is the point at which a stimulus causes pain. Previous studies in literature have examined the effect of cervical proprioception on the pressure pain threshold, although the reported results are conflicting. Some studies report a correlation between impaired proprioception and pain intensity, including the pressure pain threshold [26], in the presence of chronic pain. Cervical proprioception is often impaired in the presence of chronic pain, contributing to disturbances in sensorimotor control [24]. Studies have shown that chronic neck pain can lead to alterations in cervical motor behaviours, reduced activation of the deep cervical muscles and increased activation of superficial muscles [24]. Such imbalances affect the proprioceptive feedback from the cervical spine, leading to impaired proprioception [24]. In the present study, the effects of impairments in cervical proprioception on the pressure pain threshold in the cervical region were not significant, contrasting with the results of previous studies, although this may be attributed to the absence of neck pain in the present study participants.

In the temporal summation test applied in the present study, to measure gradual increases in pain in response to repeated stimuli, temporal summation was measured through the application of 10 consecutive pressure stimuli up to the pressure pain threshold, as determined by a pressure algometer. The participant was then asked to identify the point at which they first felt discomfort (final threshold), and the difference between the initial threshold and the final threshold was recorded as the temporal summation value.

There have been no studies to date investigating the effect of cervical proprioception on temporal summation and conditioned pain modulation in asymptomatic individuals, as studies of temporal summation and conditioned pain modulation have focused on individuals with chronic pain. These studies tend to explore the different functioning of the facilitation and inhibition mechanisms in those with such chronic conditions as chronic low back pain or fibromyalgia [39–41]. In the present study, the absence of any effect of cervical proprioception on these two parameters may be due to the absence of pain in those in our sample. The complex relationship between proprioception and pain has yet to be fully defined. As can be concluded from the results of the present study, errors related to cervical proprioception may develop earlier than alterations in cervical QST, which suggests that proprioception may develop earlier than pain, and indicates a need for further studies of this subject.

The present study has some limitations, the first of which is its inclusion only of young and asymptomatic individuals, limiting the generalisability of the findings to other populations, including older adults or those with chronic pain. Younger participants were selected to support the homogeneity of the sample, as musculoskeletal issues tend to increase with age. However, this narrow sample range may not fully capture the broader variability present in the general population. Future studies should aim to include a more diverse sample, incorporating individuals from different age groups and clinical populations, to enhance the external validity of the findings. Furthermore, the cross-sectional design of the study hindered the identification of any causal relationships between CPE and the measured outcomes. While significant associations were found, the study design does not allow us to determine whether CPE directly influences body awareness and sensory processing or whether these factors contribute to CPE. Longitudinal studies with controlled interventions are needed to establish a clearer cause-and-effect relationship. Finally, the study’s reliance on self-reported methods for the measurement of body awareness may introduce subjective bias, as these methods are inherently influenced by individual perceptions and interpretations. Self-reporting is a widely used approach for data collection in epidemiologic and medical research, requiring participants to respond to questions without researcher interference [42]. However, compared to other sources of information, such as medical records or laboratory measurements, data obtained through self-reporting is often considered less reliable and susceptible to self-reporting bias [42]. Nevertheless, when applied appropriately, self-reported data can provide a broader range of responses than many other data collection tools [43]. Therefore, integrating self-reported measures with objective assessments is recommended for future studies to enhance data accuracy and provide a more comprehensive understanding of the studied phenomenon.

Despite these limitations, the present study has several notable strengths. One of its most significant contributions is that it is the first study to evaluate the effects of CPE on body awareness. By addressing this gap in the literature, it offers a new perspective on the relationship between cervical proprioception and body awareness, enhancing our understanding of this interaction.

Additionally, this study is the first to examine the relationship between CPE and QST, establishing a connection between cervical proprioception and sensory processing mechanisms. This contribution is particularly significant, as it enables a comprehensive evaluation of the complex interplay between proprioception, the central nervous system, and pain processing mechanisms.

Finally, these findings not only contribute to theoretical knowledge but also provide a strong foundation for future research. Beyond its academic contributions, this study has the potential to serve as a key reference in the clinical management of proprioceptive dysfunctions and in clinical decision-making processes. By bridging existing gaps in the literature, this study adds a missing piece to the proprioception puzzle, offering valuable insights into body awareness and sensory processing mechanisms.

## Conclusion

This study is the first to investigate the effects of CPE on both QST and body awareness. While no significant effect was observed on QST, the findings underscore the substantial impact of CPE on body awareness, demonstrating that proprioceptive errors can impair an individual’s perception of their own body. By addressing an unexplored area in the literature, this study provides new insights into the relationship between proprioception, body awareness, and pain processing mechanisms.

Clinically, the results emphasize the need to incorporate body awareness-focused interventions into treatment protocols for patients with CPE. Overall, this study fills a critical gap in the existing knowledge and contributes to a deeper understanding of the complex interplay between proprioception, body awareness, and QST findings.

## Supporting information

S1 File Study protocol approved-without logo. (DOCX)

S2 File TREND statement checklist. (DOCX)
