# Supplementary material for: The proprioceptive puzzle: An observational study investigating the effects of cervical proprioceptive errors on quantitative sensory testing and body awareness in young individuals
Source: PLoS One. 2025 Apr 21;20(4):e0321645. doi: 10.1371/journal.pone.0321645 (PMC12011245; doi:10.1371/journal.pone.0321645)
Supplement: S1 File — (DOCX) [file pone.0321645.s001.docx]

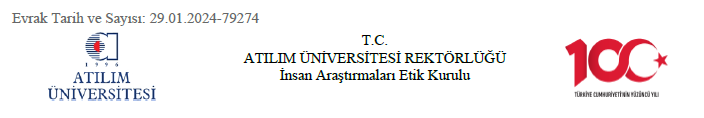


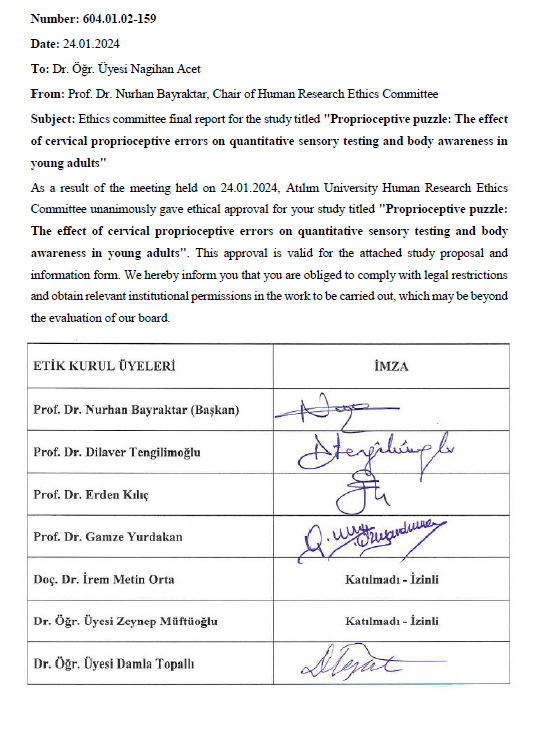


**ATILIM UNIVERSITY HUMAN RESEARCH ETHICS COMMITTEE APPLICATION DOCUMENT CHECKLIST**

After completing all the information in the application form, please check the Application Document Checklist below and include this form as the first page of your submission. Submit your application file to the Rectorate Human Research Ethics Committee Presidency.

Note: The document must be completed on a computer and printed. Handwritten documents will not be accepted.

Application Document Checklist:

✓Ethics Committee Application Form

✓Voluntary Participation Form

- Post-Participation Information Form (if applicable)
- Parental Consent Form (if applicable)

✓Copies of written data collection tools (e.g., survey, scale, test, etc.)

**Applicant Researcher:**

**Name-Surname:** Nagihan Acet

Date:22.01.1014

**Signature:**

**ATILIM UNIVERSITY HUMAN RESEARCH ETHICS COMMITTEE APPLICATION FORM**

Our university staff/students or third parties can apply to the Atılım University Human Research Ethics Committee for studies requiring the collection of information from human participants. Researchers must submit this application form and other required documents to the Atılım University Human Research Ethics Committee. Studies may only commence after receiving approval from the Ethics Committee. Student research not conducted under the Undergraduate Research Project (URP) and performed within undergraduate courses will be reviewed by the respective instructor and departments. Such studies do not require ethics committee approval.

**TITLE OF THE STUDY:** Proprioceptive puzzle: The effect of cervical proprioceptive errors on quantitative sensory testing and body awareness in young adults

**LEAD RESEARCHER:* Nagihan Acet**

Signature:

ASSISTANT RESEARCHERS:

1. Sena Begen

Signature:

INSTITUTION WHERE THE RESEARCH WILL BE CONDUCTED: Atılım University, Faculty of Health Sciences, Department of Physiotherapy and Rehabilitation

LEAD RESEARCHER: Nagihan Acet

Department: Department of Physiotherapy and Rehabilitation

Title: Asst. Prof.

Institution: Department of Physiotherapy and Rehabilitation

Phone: +90 532 205 26 05

Email:

Address: Atılım University Faculty of Health Science. Ankara, TURKEY

* In thesis research, academic advisors must also be listed as researchers.

**ASSISTANT RESEARCHERS:**

Title: Assistant Research

Name-Surname: Sena Begen

Institution: Department of Physiotherapy and Rehabilitation

**Nature of the Research:**

- Scientific Research Project ✓
- Undergraduate Research
- Master's Thesis (Thesis Advisor):
- Doctoral Thesis (Thesis Advisor):
- Other (please specify):

**Type of Research:**

- Survey Study ✓
- Observation ✓
- Scale Development Study
- File (Archive) Review
- Data Source Review
- System-Model Development Study
- Other (please specify):

**Research Support:**

- Unsupported
- Supported
- If supported, by which institution:
- University
- TUBITAK
- State Planning Organization (DPT)
- Other (please specify):

**Data Collection Period:**

Start: 02.2024

End: 01.2025

**Locations/Institutions Where Data Will Be Collected:** Department of Physiotherapy and Rehabilitation

If there are more than five locations, list them separated by commas.

**Application Status:**

- New Application ✓
- Resubmission (Project No.)
- Protocol Change
- Continuation of a Previously Approved Project (Project No.)

**Research Summary (Please explain briefly and simply):**

The term "proprioception" was first introduced in 1906 by the English neurophysiologist Charles Sherrington, derived from the Latin words "proprius" (own) and "perception." This perception allows a person to know the location of their body and/or body parts at any given moment by utilizing sensory information from receptors located in the joints, muscles, and tendons. According to Sherrington, proprioception is defined as "the perception of the position of the body or body segments in space." In this context, Sherrington referred to proprioception as the "perception of body position and movement" [1].

The word “perception,” which means “to perceive” in Turkish, is derived from the Latin term "percepio" (to perceive) and refers to the process by which humans interpret, organize, and make sense of sensory information to internalize and understand their environment [2].

All perceptions require signals within the nervous system that originate from the physical stimulation of various sensory organs [3]. For example, hearing involves sound waves affecting the eardrum; vision involves light striking the retina of the eye, with these forms of energy being converted into electrical signals within neurons. Similarly, proprioception requires a stimulus that leads to the activation of mechanoreceptors through body movements (changes in body position). However, perception is not just the passive reception of a sensory signal but also shaped by memory and learning [4]. In this understanding, proprioception can be defined as an individual's ability to integrate sensory signals from mechanoreceptors, enabling them to determine the position and movement of body segments in space. As a result, a person’s proprioceptive performance depends on both the adequacy of the available proprioceptive information (hardware-peripheral receptors) and the individual's ability to process proprioceptive information (central processing-software) [5].

Proprioception plays a fundamental role in human movement control during daily activities, exercise, and sports. In the literature, it is more frequently addressed in the context of injury or the presence of pain. Numerous studies report that proprioception changes in the presence of pain. However, it is unclear whether proprioceptive deviation predisposes to pain or if the presence of pain reduces proprioception. Therefore, assessing proprioceptive deficits in healthy individuals without pain could shed light on the "chicken or the egg" dilemma regarding proprioception and pain.

Additionally, previous studies have predominantly focused on proprioceptive deviations in extremities, such as the ankle and knee, while overlooking spinal proprioception. However, cervical proprioception is particularly important for postural stability [6]. Due to the abundance of mechanoreceptors in the suboccipital muscles, the cervical region is identified as a primary source of proprioceptive information [7]. While one gram of muscle tissue in the suboccipital region contains 200 muscle spindles, one gram of the first lumbrical muscle in the thumb contains only 16 spindles [8, 9]. The cervical muscles, especially M. longus capitis, M. longus colli, M. obliquus capitis superior, M. obliquus capitis inferior, M. rectus capitis posterior major, and M. rectus capitis posterior minor, which are rich in muscle spindles, provide a continuous flow of information to the central nervous system and regulate muscle activation based on this information [10].

Furthermore, the receptors in the upper cervical region are connected to the central nervous system, vestibular system, and visual system [11, 12]. The information obtained from these receptors is involved in key reflexes responsible for head, eye, and postural stability, including the cervico-collic reflex, cervico-ocular reflex, and tonic neck reflex [13].

Considering the central nervous system's connections with proprioception, even in the absence of injury or pain, deviations in cervical proprioception in healthy individuals could lead to changes in pain-related parameters within the central nervous system. Quantitative Sensory Testing (QST) is a psychophysical method used to assess pain perception and evaluate the presence of peripheral and central sensitization. These tests are widely used to evaluate pain perception in patients with musculoskeletal conditions, such as spinal cord injuries, back pain, whiplash, and osteoarthritis [14-16]. Pressure pain threshold, temporal summation, and conditioned pain modulation are three common types of QST measures used to diagnose altered pain processing mechanisms [17]. These tests are performed using various modalities such as thermal or mechanical stimuli [17]. Pain thresholds are defined as the point at which a sensation (e.g., pressure) becomes painful [17], while temporal summation refers to a gradual increase in pain in response to repeated stimuli, such as heat or pressure [18]. Conditioned pain modulation, a key mechanism of endogenous analgesia, evaluates the potential for pain perception to be reduced by applying mechanical, electrical, thermal, or cold stimuli to a different area. Tests such as pressure pain thresholds, cold or heat pain thresholds, conditioned pain modulation, and temporal summation provide insights into the functioning of A and C fibers and their central pathways. They can detect both peripheral sensitization and centrally mediated symptoms (e.g., secondary hyperalgesia and temporal summation) [17, 19, 20]. Therefore, QST can be useful in understanding an individual’s pain mechanisms, which can inform a more personalized approach to rehabilitation and pain management.

The impact of proprioception on central processing is a crucial topic that needs further attention. However, the effect of proprioceptive deviation on central mechanisms remains uncertain. Proprioceptive deviation may lead to changes in the central nervous system’s pain processing mechanisms. To our knowledge, there is no study in the literature investigating the effect of deviations in cervical proprioception on QST in healthy individuals without trauma or pain. This study will add significant value to the literature in this regard.

In recent years, the term "body awareness" has emerged as a key concept in health-related scientific studies. In its most basic sense, body awareness refers to an individual’s awareness of their body parts or dimensions. From a neuroscience perspective, body awareness is the brain’s recognition of messages received from other parts of the body and the external environment. The messages a person receives encompass not only their body and movements (intra-corporeal awareness) but also the characteristics and locations of external objects (extra-corporeal awareness). Over time, these messages are integrated to form the individual’s experiences. This knowledge and experience are crucial in understanding, interpreting, and forming social interactions with the environment. Therefore, body awareness is the awareness of one’s own body and the characteristics of external objects [21].

An individual’s ability to process proprioceptive information, known as central processing, may be directly related to body awareness. However, to our knowledge, there is no study in the literature investigating the effect of cervical proprioceptive errors on body awareness. We believe that this study will shed light on this aspect and contribute to the literature.

In light of the above information, determining the impact of proprioceptive deviations on QST and body awareness in young, healthy individuals could significantly contribute to the development of preventive physiotherapy approaches and new treatment algorithms. The findings could pave the way for developing customized physiotherapy protocols for clinical use. Additionally, this research may serve as a valuable source of information for future healthcare strategies by evaluating the potential contribution of cervical proprioceptive deviations to pain and movement-related issues in young adults. By addressing specific interactions within the body-movement-pain complex, our study will also help better understand how cervical proprioceptive deviations shape this complex in young adults.

**Objective:** The aim of the present study is to reveal the impact of cervical proprioceptive deviations on pressure pain threshold, temporal summation, conditioned pain modulation, and body awareness in young healthy individuals.

**Methodology:**

Study Design: A prospective cross-sectional study will be conducted after obtaining approval from the Atılım University Ethics Committee with the approval date and reference number provided. After being informed about the study, participants who voluntarily agree to participate will be included by signing a written consent form.

Participants:
Participants will be included in the study through simple randomization. Each participant will be evaluated face-to-face only once. Between January 2024 and January 2025, individuals who meet the inclusion criteria and volunteer to participate will be included. The participants will be selected from staff members on the Atılım University campus. Participants will be invited to the study through a recruitment poster containing details of the study, which will be posted on the announcement page of Atılım University. The evaluations will be conducted face-to-face by Research Assistant Sena Begen in the research laboratory of the Department of Physiotherapy and Rehabilitation at Atılım University, located on the same campus where the participants work.

In this study involving young, healthy participants, cervical proprioception will be assessed using a head position error test with a CROM device in right and left rotation directions. Based on the presence of proprioceptive deviation (cut-off: >5°), participants will be divided into two groups: the experimental group (those with a proprioceptive deviation greater than 5°) and the control group (those with a proprioceptive deviation less than 5°). Participants with a deviation greater than 5° in at least one direction will be included in the experimental group with proprioceptive deficits.

In both groups, body awareness will be assessed using the Body Awareness Questionnaire; pressure pain threshold and temporal summation will be measured bilaterally using an algometer device (2 cm lateral to the C2 and C7 spinous processes, midpoint of the upper part of the trapezius muscle, and over the temporomandibular joint).

Inclusion Criteria:
Individuals aged 18-25 who voluntarily agree to participate will be included.

Exclusion Criteria:
Exclusion criteria will include sensory loss, the presence of neck pain, radicular pain, cervical or upper extremity surgery, a history of cervical trauma, neurological diseases, musculoskeletal injuries, vestibular pathology, and cold allergy.

Data Collection:

Sociodemographic Characteristics Evaluation: Participants' age, height, weight, body mass index, presence of any chronic disease, and medications will be recorded on an evaluation form.

Cervical Proprioception Assessment: The "head position error test" will be used to assess cervical proprioception. Based on the presence of proprioceptive deviation (cut-off: >5°), participants will be divided into two groups: the experimental group (with a proprioceptive deviation greater than 5°) and the control group (with a proprioceptive deviation less than 5°). Participants with a deviation greater than 5° in at least one direction will be included in the experimental group with proprioceptive deficits.

The head position error test will be evaluated using a CROM device in two directions, right and left rotation, while the participant is seated [22]. The maximum range of cervical joint motion will be determined. The midpoint of the maximum range will be defined as the "target position." With their eyes closed, participants’ heads will be passively moved to the target position by the physiotherapist, held for 3 seconds, and then returned to the neutral position. The participant will then be asked to actively return their head to the same target position, and this movement will be repeated six times. The differences between the target position and the obtained position will be recorded, and their averages calculated. A deviation greater than 5° will be recorded as a proprioceptive deficit.

Body Awareness Assessment: The body awareness questionnaire consists of 18 items divided into 4 subgroups (1. Changes in body processes, 2. Sleep-wake cycle, 3. Prediction of illness onset, 4. Prediction of body reactions), aiming to determine normal or abnormal sensitivity levels. Participants rate each item on a scale from 1 to 7. The total score is calculated, with a higher score indicating better body awareness. The validity and reliability of the questionnaire have been reported as high [23].

Pressure Pain Threshold Assessment: A pressure algometer (Baseline Force Gauge Model 12–0304; Baseline, NY, USA) will be used to assess the pressure pain threshold. A force will be applied perpendicularly to a point with an area of 0.5 cm² at approximately 3 N/s. While the patient is seated, pressure will be applied bilaterally using the algometer to different points in the head and neck region (2 cm lateral to the C2 and C7 spinous processes, midpoint of the upper part of the trapezius muscle, and over the temporomandibular joint).

In the test protocol, digital pressure will be applied vertically to the selected points, and the pressure will be recorded. The participant will be asked to indicate the first point at which they feel discomfort. The pressure value at that moment will be read from the device and recorded as the "pressure pain threshold." Two measurements will be taken at each site, and the average will be calculated. The total score will be calculated by averaging all values.

Temporal Summation Assessment: Ten minutes after the pressure pain threshold measurement, temporal summation will be assessed bilaterally at the same points (2 cm lateral to the C2 and C7 spinous processes, midpoint of the upper part of the trapezius muscle, and over the temporomandibular joint). The participant will receive 10 consecutive applications of pressure at the “pressure pain threshold” (the first point of discomfort). Afterward, the participant will be asked to identify the point where they feel discomfort again (final threshold). The difference between the initial and final thresholds will be recorded as the temporal summation value [24].

Conditioned Pain Modulation Assessment: In the first step of the assessment, increasing pressure will be applied with the algometer to the right trapezius until the participant reports a pain intensity of 4/10 on the Visual Analog Scale. The participant will be asked to indicate when they feel 4/10 pain, and the stimulus will be stopped at that point. Five minutes after the test stimulus, the participant will be asked to immerse their right hand in ice-cold water (7°C) for 20 seconds as the conditioned stimulus. For each application, 3 liters of tap water will be poured into a glass container, and ice cubes will be used to maintain the temperature at 7°C, which will be verified with a thermometer. Immediately after removing the hand from the water, the same pressure as the 4/10 pain level on the right trapezius will be applied to the left trapezius with the algometer, and the participant will be asked to rate the pain again. For participants unable to keep their hand in the water for 20 seconds, the evaluation will be performed as soon as the hand is removed from the water. The ratio of the pain ratings before and after the conditioned stimulus will be multiplied by 100 to calculate the final score [25].

Sample Size Calculation: The required sample size for the study was calculated using the GPower program after the pilot study. The required number of participants for 95% power, with an effect size of 5%, was determined to be 24 per group, for a total of 48 participants. To account for a potential 20% dropout rate, a total of 58 participants are planned to be included.

Statistical Analysis: Analyses will be performed using IBM SPSS Statistics 23 software. Descriptive statistics (mean, standard deviation) will be provided for numerical variables, and frequencies (number, percentage) will be given for categorical variables.

The normality assumptions of numerical variables across groups will be assessed using the Shapiro-Wilk or Kolmogorov-Smirnov normality tests, depending on the data. If the data are normally distributed, parametric methods will be used; if not, non-parametric methods will be applied. Independent sample t-tests will be used for normally distributed data, and the Mann-Whitney U test will be applied for non-normally distributed data.

**Expected Outcomes:**

- The pain thresholds of young healthy individuals with cervical proprioceptive deviation are different/similar compared to those without proprioceptive deviation.
- The temporal summation values of young healthy individuals with cervical proprioceptive deviation are different/similar compared to those without proprioceptive deviation.
- The conditioned pain modulation in young healthy individuals with cervical proprioceptive deviation is different/similar compared to those without proprioceptive deviation.
- The body awareness of young healthy individuals with cervical proprioceptive deviation is different/similar compared to those without proprioceptive deviation.

**Selected References:**

1. Sherrington, C., *The integrative action of the nervous system*. 1952: CUP Archive.

2. Schacter, D.L., D.T. Gilbert, and D.M. Wegner, *Introducing psychology*. 2009: Macmillan.

3. Goldstein, E.B., *Sensation and perception*. 1989: Wadsworth/Thomson Learning.

4. Bernstein, D.A. and P.W. Nash, *Essentials of psychology*. 2008: Houghton Mifflin Company.

5. Han, J., et al., *Assessing proprioception: a critical review of methods.* Journal of Sport and Health Science, 2016. 5(1): p. 80-90.

6. Humphreys, B.K., *Cervical outcome measures: testing for postural stability and balance.* Journal of manipulative and physiological therapeutics, 2008. 31(7): p. 540-546.

7. Kulkarni, V., M. Chandy, and K. Babu, *Quantitative study of muscle spindles in suboccipital muscles of human foetuses.* Neurology India, 2001. 49(4): p. 355.

8. Boyd-Clark, L., C. Briggs, and M. Galea, *Muscle spindle distribution, morphology, and density in longus colli and multifidus muscles of the cervical spine.* Spine, 2002. 27(7): p. 694-701.

9. Liu, J.-X., L.-E. Thornell, and F. Pedrosa-Domellöf, *Muscle spindles in the deep muscles of the human neck: a morphological and immunocytochemical study.* Journal of Histochemistry & Cytochemistry, 2003. 51(2): p. 175-186.

10. Silva, A.G. and A.L. Cruz, *Standing balance in patients with whiplash-associated neck pain and idiopathic neck pain when compared with asymptomatic participants: A systematic review.* Physiotherapy theory and practice, 2013. 29(1): p. 1-18.

11. Grace Gaerlan, M., et al., *Postural balance in young adults: the role of visual, vestibular and somatosensory systems.* Journal of the American Academy of Nurse Practitioners, 2012. 24(6): p. 375-381.

12. Corneil, B.D., E. Olivier, and D.P. Munoz, *Neck muscle responses to stimulation of monkey superior colliculus. I. Topography and manipulation of stimulation parameters.* Journal of Neurophysiology, 2002. 88(4): p. 1980-1999.

13. Peterson, B.W., *Current approaches and future directions to understanding control of head movement.* Progress in brain research, 2004. 143: p. 367-381.

14. Klyne, D.M., et al., *Are signs of central sensitization in acute low back pain a precursor to poor outcome?* The journal of pain, 2019. 20(8): p. 994-1009.

15. Boakye, M., et al., *Quantitative testing in spinal cord injury: overview of reliability and predictive validity.* Journal of neurosurgery: Spine, 2012. 17(Suppl1): p. 141-150.

16. Fingleton, C., et al., *Pain sensitization in people with knee osteoarthritis: a systematic review and meta-analysis.* Osteoarthritis and cartilage, 2015. 23(7): p. 1043-1056.

17. Cruz-Almeida, Y. and R.B. Fillingim, *Can quantitative sensory testing move us closer to mechanism-based pain management?* Pain medicine, 2014. 15(1): p. 61-72.

18. Nie, H., T. Graven-Nielsen, and L. Arendt-Nielsen, *Spatial and temporal summation of pain evoked by mechanical pressure stimulation.* European journal of pain, 2009. 13(6): p. 592-599.

19. Woolf, C.J., *Central sensitization: implications for the diagnosis and treatment of pain.* pain, 2011. 152(3): p. S2-S15.

20. Vardeh, D., R.J. Mannion, and C.J. Woolf, *Toward a mechanism-based approach to pain diagnosis.* The Journal of Pain, 2016. 17(9): p. T50-T69.

21. Berlucchi, G. and S.M. Aglioti, *The body in the brain revisited.* Experimental brain research, 2010. 200: p. 25-35.

22. Revel, M., C. Andre-Deshays, and M. Minguet, *Cervicocephalic kinesthetic sensibility in patients with cervical pain.* Archives of physical medicine and rehabilitation, 1991. 72(5): p. 288-291.

23. Mehling, W.E., et al., *Body awareness: construct and self-report measures.* PloS one, 2009. 4(5): p. e5614.

24. Heisler, A.C., et al., *Association of pain centralization and patient‐reported pain in active rheumatoid arthritis.* Arthritis care & research, 2020. 72(8): p. 1122-1129.

25. Yarnitsky, D., et al., *Recommendations on practice of conditioned pain modulation (CPM) testing.* European journal of pain, 2015. 19(6): p. 805-806.

**Does the research require providing biased/misleading information to participants or keeping the study's purpose confidential?**

- Yes
- No✓

If yes, please explain:

**Are there any questions in the research that could pose a risk to the physical or mental health of the participants?**

- Yes
- No✓

If yes, please explain:

**Are the participants' private information protected in the research?**

- Yes✓
- No

If yes, please explain: The collected data will be stored in Excel and SPSS files on a computer accessible only by the researchers who have the password. As stated in the consent form, no one other than the researchers will have access to this information. Personal data obtained during the assessments in the study will not be shared with any institution or individual.

**Does the research involve minors or disabled individuals?**

- Yes
- No✓

If yes, please explain:

**Is clear and understandable information provided to the participants about the research? Please explain:** At the beginning, the purpose and content of the research will be explained in detail to the participants by the responsible researcher, and a written informed consent form will be prepared for the study. After carefully reading this form, the signatures of those who agree to participate in the study will be obtained. The interventions to be performed on the participants will be explained in the consent form using clear and understandable terminology as much as possible.

**Have the conditions for participants' voluntary participation and withdrawal from the study been clearly defined? Please explain:**

The conditions for the participation and withdrawal of volunteers from the study are specified in the inclusion and exclusion criteria.

**Expected Number of Participants: 58**

**Please mark the options that best describe your participants (multiple options can be selected):**

- Preschool Children
- Primary School Students
- High School Students
- University Students✓
- Child Workers
- Employed Adults✓
- Unemployed Adults✓
- Males✓
- Females✓
- Elderly
- Individuals with Intellectual Disabilities
- Individuals with Physical Disabilities
- Prisoners
- Other (please specify):

**Please mark the procedures that will be used in the study:**

Survey/Questionnaire✓

Interview

Observation✓

Computerized Application

Video Recording

Audio Recording

Other (please specify):

PLEASE SIGN ALL PAGES OF THE APPLICATION FORM!
